# Finite Element Analysis of Anterior Odontoid Screw Fixation for Type II Odontoid Fractures

**DOI:** 10.3390/ma19040825

**Published:** 2026-02-23

**Authors:** Pedro Miguel González-Vargas, Antía Millán, José Luis Thenier-Villa, Aida Badaoui, Cesáreo Conde, Juan Pou, Antonio Riveiro

**Affiliations:** 1 Department of Neurosurgery, University Hospital Complex of Vigo, 36312 Vigo, Spain; 2 Neuroscience Research Group, Galicia Sur Health Research Institute, 36312 Vigo, Spain; 3 LaserON Research Group, CINTECX, University of Vigo, 36310 Vigo, Spain; 4 Department of Neurosurgery, University Hospital of Lérida, 25198 Lérida, Spain; 5 Materials Engineering, Applied Mechanics and Construction Department, E.E.I., University of Vigo, 36310 Vigo, Spain; 6 Galicia Sur Health Research Institute (IIS Galicia Sur), SERGAS—Universidade de Vigo (UVIGO), 36310 Vigo, Spain; 7 Applied Physics Department, E.E.I., Universidade de Vigo, 36310 Vigo, Spain

**Keywords:** odontoid, type II fracture, screw fixation, FEM analysis

## Abstract

*Introduction:* Type II odontoid process fractures are common in the adult population, and anterior screw fixation aims to restore C1–C2 complex stability while preserving cervical motion. This study focuses on the numerical analysis of odontoid fractures, evaluating the structural behavior after anterior screw fixation using finite element simulations. *Methods:* Forty-eight patients (males, females, 74 years old on average) diagnosed with type II odontoid fractures and treated surgically between 2015 and 2023 were included in the study. Various loading conditions (magnitude and direction) were simulated to analyze displacements and stress distributions after screw insertion. *Results:* Screw fixation significantly fixes fractured vertebrae, but stress and deformation are considerably larger than in unfractured cases. Posterior oblique loads produced the highest stress concentrations, particularly at the base of the odontoid and the screw-bone interface. Male models exhibited greater total deformations and stresses under the same loading conditions, suggesting relevant biomechanical differences based on sex. *Conclusions:* Anterior odontoid screw fixation provides effective stabilization in type II odontoid fractures, although its performance depends on factors such as load vector and patient-specific anatomical characteristics. These findings support the use of FEM simulation as a valuable tool for personalized surgical analysis.

## 1. Introduction

**Figure 1:**
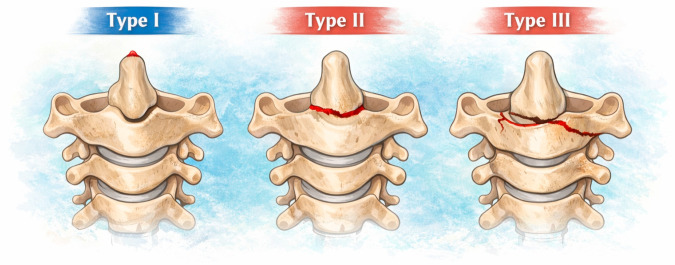
Anderson and D’Alonzo’s classification system of dens fractures. Type I: fracture trace affecting the dens exclusively. Type II fracture trace runs along the dens base. Type III fracture compromises the C-2 body, and it may compromise the articular surface. Arrow = fracture trace.

Fractures of the odontoid process are among the most frequent injuries of the upper cervical spine, accounting for up to 15–20% of all cervical fractures, and over 50% of axis (C2) fractures in older adults [1,2]. These injuries are particularly associated with low-energy falls in geriatric patients, due to progressive loss of bone mineral density and postural control disorders [3,4,5]. Odontoid fractures are also relevant in younger adults during motor vehicle crashes, especially during frontal impacts [6]. The most widely used classification in odontoid fracture is that of Anderson and D’Alonzo, which distinguishes three main types [7] (see Figure 1). Among them, type II—located at the base of the odontoid process—is the most prevalent and also the most unstable, with pseudoarthrosis rates exceeding 30% in the absence of surgical treatment [8,9].

Several surgical techniques have been developed to manage this fracture, with anterior fixation using a cannulated screw being one of the most commonly employed in selected patients [10]. This technique allows direct stabilization of the fracture site while preserving physiological movement of the atlantoaxial complex. Its benefits include lower morbidity compared to posterior techniques and the possibility of a faster functional recovery [11]. However, the biomechanical efficacy of this method may be affected by multiple anatomical and biomechanical factors, such as the orientation of the fracture line, screw insertion angle, bone density, and the forces acting on the cervical spine during daily activities [12].

In this context, computational finite element models (FEM) have emerged as a very practical and powerful way for evaluating the efficacy of medical treatments and an essential tool for the biomechanical analysis of complex anatomical structures such as the cervical spine [13,14]. These models enable the simulation of specific clinical conditions, assessment of system responses under different loads, and objective comparison of various surgical strategies [15,16]. Unlike cadaveric studies, FEM models offer reproducibility, control over variables, and adaptability to realistic clinical conditions. In addition, they are more cost-effective compared to traditional experimental methods [17]. In spinal surgery, they have proven useful for predicting implant failures, evaluating stress distributions, and optimizing surgical procedures [18,19].

Scott Graham et al. performed a numerical FE model to study stress patterns, predict fracture patterns, and transitions between Type II and Type III odontoid fractures in a model of a C-2 vertebra produced from CT data. Loads in a range of 1279–1736 N and different application directions (inclination angles of −45° to 15° and 0° to 45° in the coronal and horizontal planes, respectively) were applied. This model is able to capture the transition between Types II and III and agrees with experimental data [20]. Similar conclusions were obtained by Yuan et al. in another study using smaller loads (200 N) [21]. In a more recent work, Güvercin et al. also used a numerical model to study the tension and deformation of the odontoid in patients with ponticulus posticus variation, under different neck motion conditions [22]. González-Vargas et al. developed a numerical FE model to analyze the influence of different loads on a population sample consisting of 52 patients and grouped by sex and age. It was found that differences between sexes are much more prominent for older than for younger patients [13]. Maximum stresses are higher in the odontoid of males than females.

Li et al. performed an FE model of the odontoid fracture, including the inferior extremity of the occipital bone, and the C1–C3 vertebral body. They analyzed the biomechanical stability of four self-designed C0–C3 transarticular screw and rod fixation techniques, and determined the most efficient ones by analyzing the displacement and stress maps determined using the numerical model [23]. A similar approach was also followed by Hu et al. to study the efficacy of an artificial atlantoodontoid joint (AAOJ) replacement [24]. The results indicated that this technique can be an alternative to stabilize a type II odontoid fracture injury. Liu et al. developed a numerical model of the upper cervical region of a healthy male volunteer to study the stability of three-different fixation methods used to treat the combination of atlas–axis fractures [25]. The model included the vertebral body, disc, facet, and major ligaments. Three surgical fixation methods, such as the atlantoaxial pedicle screws fixation, occipital–cervical fusion, and transarticular screw fixation techniques were studied. This study concludes that all the methods limit the motion of the C1-C2 articulation and provide the required stability to promote the fusion. Paksoy et al. also developed a numerical model of the upper cervical region of a healthy male volunteer to compare the stability when using single and double anterior odontoid screw fixation techniques [26]. A reduction in the implant failure when using a double screw fixation was observed. In these last two studies, the fractures were simulated.

In view of the previous works, and to the best of the authors’ knowledge, a comprehensive numerical study of type II odontoid fractures treated with an anterior odontoid screw (one of the most common surgical treatment methods) under varied mechanical conditions (load magnitude and direction) is missing in the literature. Then, in this work, we have studied, using a FEM approach, the fracture treated with an anterior odontoid screw, aiming to quantify its protective effect under varying mechanical conditions. In contrast to previous works, we have studied surgically treated models. Additionally, comparisons between male and female models are incorporated, based on morphological and densitometric differences that may influence biomechanical response.

In this work, several modeling simplifications were adopted—such as homogeneous isotropic material properties and simplified contact conditions, including the omission of ligaments, support on the intervertebral disc, etc.—to reduce model complexity. However, the primary objective of this study is a comparative evaluation of anterior odontoid screw fixation under varied loading magnitudes and directions. Accordingly, the results should be interpreted in a relative sense, focusing on trends and differences between loading scenarios and patient-specific models rather than on absolute stress or deformation values. The final goal is to provide quantitative evidence to support clinical selection criteria and to contribute to the development of safer and more personalized surgical strategies.

## 2. Materials and Methods

### 2.1. Population and Image Acquisition

After approval by the Ethics Committee for Research with Medicines (CEIm) Hospital Álvaro Cunqueiro—SERGAS (Approval Reference Number: CEIm-SERGAS-2025/017) and the signing of informed consent forms by the patients a retrospective study was conducted with a sample of 48 adult patients diagnosed with type II odontoid fractures, treated surgically via anterior fixation with a cannulated screw between 2015 and 2023. All patients were treated at two hospitals in southern Galicia, and surgeries at each center were performed by the same two surgeons. Inclusion criteria were: age over 18 years, type II fracture confirmed by computed tomography (CT), and availability of DICOM-format images obtained in supine position with neutral cranial alignment. Pediatric patients and those with pathological fractures due to infectious, neoplastic, or metabolic diseases were excluded.

The mean patient age was 74 years (SD ± 9.6), with a sex distribution of 28 men (58%) and 20 women (42%). This distribution is consistent with literature reports showing an increased prevalence of these fractures in elderly patients, associated with osteopenia and low-energy trauma mechanisms [5].

### 2.2. Segmentation and 3D Modeling

CT images were processed using *3D Slicer* software (v4.11) for the segmentation of cortical and trabecular bone [27]. An optimized Hounsfield unit threshold was applied to isolate bony structures, and segmentation was subsequently refined through manual editing.

**Figure 2:**
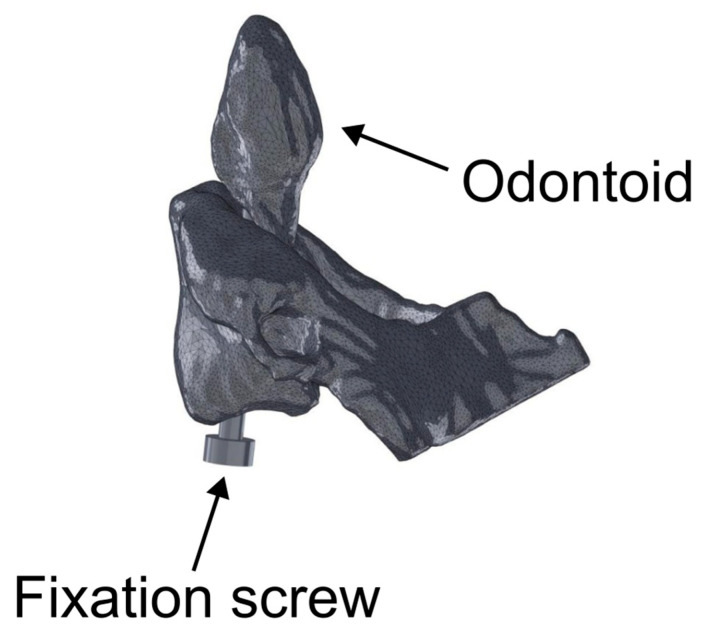
Schematic representation of a representative fractured vertebra (C2-axis), assembled with an anterior fixation screw.

The resulting 3D models were exported in STL format and further cleaned, smoothed, and converted into solid meshes using Meshmixer (v3.5.0, Autodesk, San Francisco, CA, USA) and SolidWorks 2024 (Dassault Systèmes SolidWorks Corp., Waltham, MA, USA). The final geometry includes a representation of the cannulated screw inserted along the anterior midline of the odontoid process, following standard surgical trajectory: entry at the anterior surface of the axis body and exit at the apex of the odontoid process (see a representative model in Figure 2).

The finite models were constructed by first aligning all individual geometries using anatomical landmarks to ensure consistent spatial orientation. Morphometric parameters were extracted from each model, and a representative male and female geometry was reconstructed using the mean values of the measured anatomical dimensions. This approach generated sex-specific averaged models that preserved anatomical proportions while reducing interindividual variability.

### 2.3. FEM Model Preparation

Finite element simulations were conducted using ANSYS Workbench 2023 R1 (ANSYS, Inc., Canonsburg, PA, USA). A tetrahedral mesh with adaptive refinement in regions of interest (fracture site and screw path) was used, achieving a resolution between 0.4 and 1 mm. Mesh quality was validated through a convergence analysis, evaluating the stability of maximum displacements with different element sizes. A variation of less than 3% between successive refinements confirmed the adequacy of the final mesh (see Table S1 in Supplementary Information).

To simplify the simulations, all the materials were considered homogenous, isotropic, and average properties of cortical and trabecular bone were used (percentage of volume occupied by each phase −80% cancellous bone, 20% cortical bone- was considered to obtain an average value).

Bone tissue was modeled as a homogeneous, isotropic, linear elastic material, an assumption commonly adopted in exploratory finite element analyses, but which neglects the intrinsic anisotropy, hierarchical structure, and adaptive remodeling capacity of bone. More advanced continuum formulations accounting for orthotropy, substructural evolution, and mechanically driven remodeling have been proposed in the literature and provide a more comprehensive description of bone mechanobiology, although at the expense of increased model complexity and computational cost [28,29]. Despite the modeling simplifications, the proposed approach allows the evaluation of relative differences between untreated and anterior odontoid screw–treated cases in both male and female patient-specific models.

On the other hand, the screws were considered to be composed of Ti6Al4V ELI (Grade 23) alloy. The mechanical properties assigned to each material are summarized in Table 1.

For lateral mass screws, we use a model with a 3.5 mm diameter and 40 mm length. For the odontoid screw, we use a model with a 4 mm diameter and 10 mm length. These sizes are the most used in routine practice.

### 2.4. Boundary Conditions and Applied Loads

Axis (C2) was constrained by fixing its bottom surface; then, the displacements of this surface are restricted. The occipital condyle was bonded to the axis through the fixation screw. Contacts between condyle, axis, and screw (i.e., condyle-screw, condyle-axis, and screw-axis) were simplified as bonded contacts.

**Figure 3:**
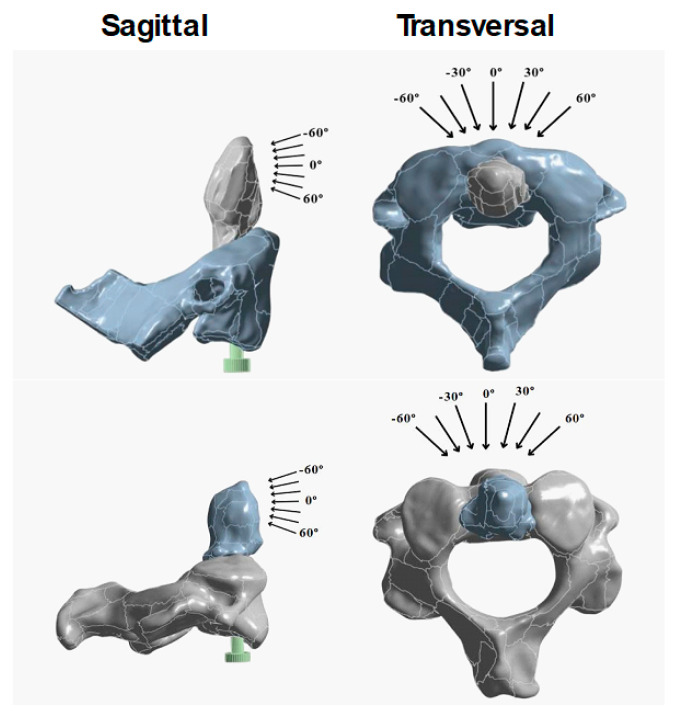
Schematic representation of the forces (plane and direction of application) for representative male (**top**) and female (**bottom**) vertebrae.

Loads of 750, 1500, 2250, and 3000 N were applied to the occipital condyle in three directions: pure vertical (0°), posterior oblique (30–60°), and left lateral oblique (30–60°), replicating conditions that simulate various loading mechanisms or physiological stresses (see Figure 3 and Figure S1). Such high loads were selected as in some experimental studies, fracture loads in a range of ≈580–1580 N were observed to produce the failure of the odontoid in an oblique configuration (45° from the sagittal plane) [33].

Three experimental conditions were evaluated for each load combination: models without a screw (control), male models with a screw, and female models with a screw.

The fracture was modeled at the base of the odontoid process, horizontally, at the neck of the process. This refers to the characteristic and most frequent fracture pattern in these cases.

### 2.5. Evaluated Parameters

After the simulation, total deformation (mm) and stresses (MPa) were determined, namely:

- Deformation: relative displacements between the atlas (C1) and the axis (C2) in the anteroposterior direction. The average total deformation in the odontoid was obtained by averaging the stress values computed by the finite element solver over the odontoid region.
- Stresses: Von Mises stress distribution in the screw, cortical bone, and fracture zone. The average stress in the odontoid was obtained by averaging the stress values computed by the finite element solver over the odontoid region.

Results were represented using color maps, and quantitative comparisons were performed.

## 3. Results

### 3.1. Influence of the Magnitude of the Force

**Figure 4:**
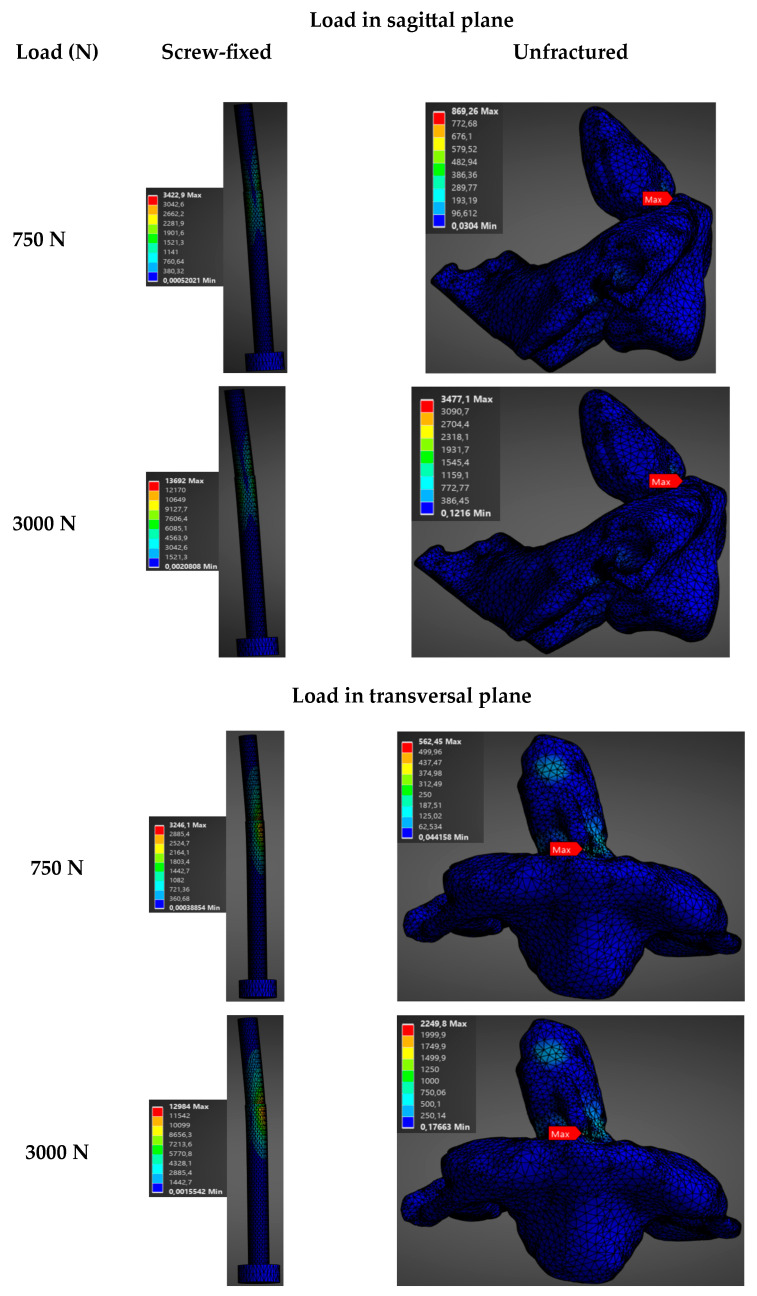
Stress distribution (MPa) as a function of applied force in the sagittal plane (load applied at 0°) and the transverse plane (load applied at −60°) for the unfractured and screw-fixed vertebrae. In the screw-fixed configuration, only the screw is shown, as the stress is entirely borne by this component.

**Figure 5:**
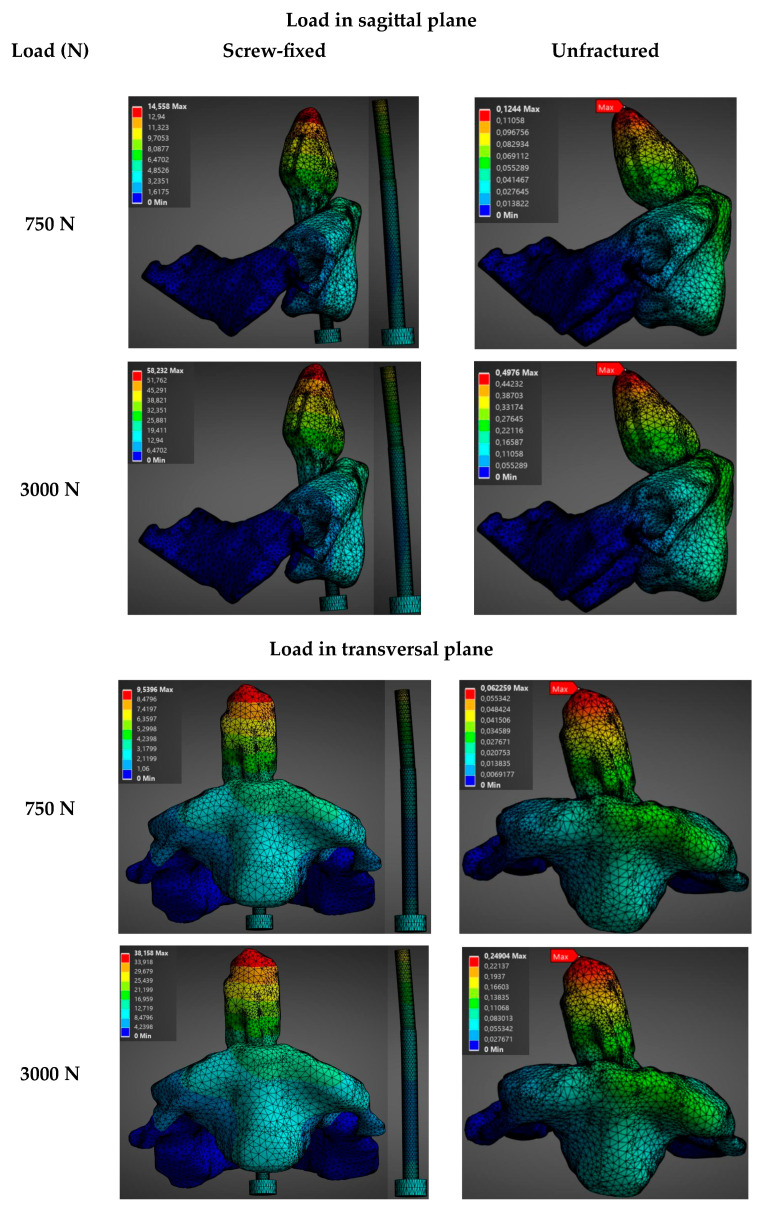
Distribution of total deformation (mm) as a function of applied force in the sagittal plane (load applied at 0°) and the transverse plane (load applied at −60°) for the unfractured and screw-fixed vertebrae.

The biomechanical behavior of the construct was analyzed as a function of the load (750 N, 1500 N, 2250 N, and 3000 N) in the sagittal (direction of application 0°) and transversal planes (direction of application −60°) for a male model. Results show a direct proportional relationship between the magnitude of the applied axial load and the evaluated variables (total deformation and stress). These are summarized in Figure 4, Figure 5 and Figure S2, which show the evolution of stress and total deformation as a function of the increment of the magnitude of the load.

As the load increases from 750 N to 3000 N, a progressive increment was observed in the intervertebral displacement between C1 and C2, as well as in the Von Mises stresses, in both the bone and the screw. As noticed in Figure 4, the stresses in the screw-fixed vertebra are totally absorbed by the screw. Moreover, Figure 4 and Figure S2a show that the average stresses are always larger in the screw-fixed than in the normal, healthy (unfractured) vertebra, irrespectively of applying a load in the sagittal or in the transversal plane.

It is also observed that the average stresses in the odontoid, both in the healthy and in the screw-fixed cases, are slightly larger when the load is applied in the sagittal than in the transversal plane. This can be related to geometrical considerations of the odontoid’s shape. The shape of the odontoid gives a larger section modulus in one plane than in another.

Regarding the deformations, similar conclusions can be drawn. As noticed in Figure 5 and Figure S2b, the deformations in the vertebra fixed with the screw are considerably higher than in the healthy case. Note that the total deformation values reported in this study include both the deformation associated with bending of the odontoid or the fixation screw (depending on whether the unfractured or screw-fixed case is being analyzed) relative to the vertebra, and the deflection arising from the torsional deformation of the vertebra, which provides with an additional displacement of the odontoid, as shown in Supplementary Videos S1 and S2. Consequently, the total deformation values reported here are comparatively high, as they reflect the combined effects of these mechanisms rather than deformation resulting solely from the bending of the odontoid or the screw relative to the vertebra.

### 3.2. Influence of the Orientation of the Load

In this case, a fixed load of 1500 N was applied in different directions (−60°, −30°, 0°, 30°, 60°) in both the sagittal and transversal planes for a screw-fixed and a healthy vertebra of a male model.

**Figure 6:**
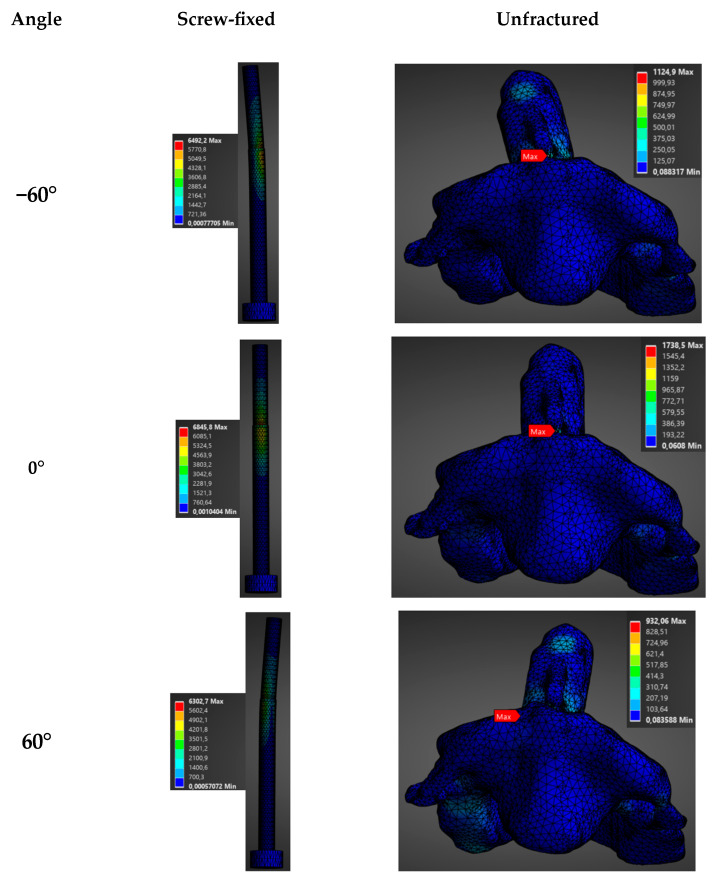
Distribution of stress (MPa) as a function of the load application angle (1500 N) in the transverse plane for the unfractured and screw-fixed vertebrae.

**Figure 7:**
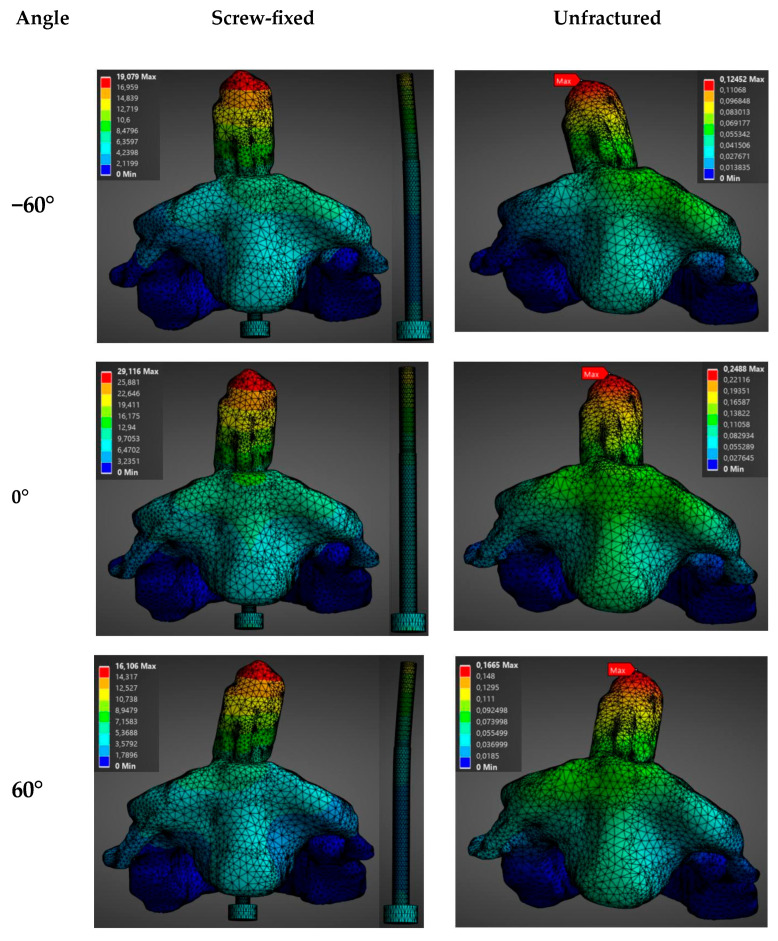
Distribution of total deformation (mm) as a function of the angle of application of a 1500 N load in the transverse plane, for both the unfractured vertebra and the screw-fixed configuration.

**Figure 8:**
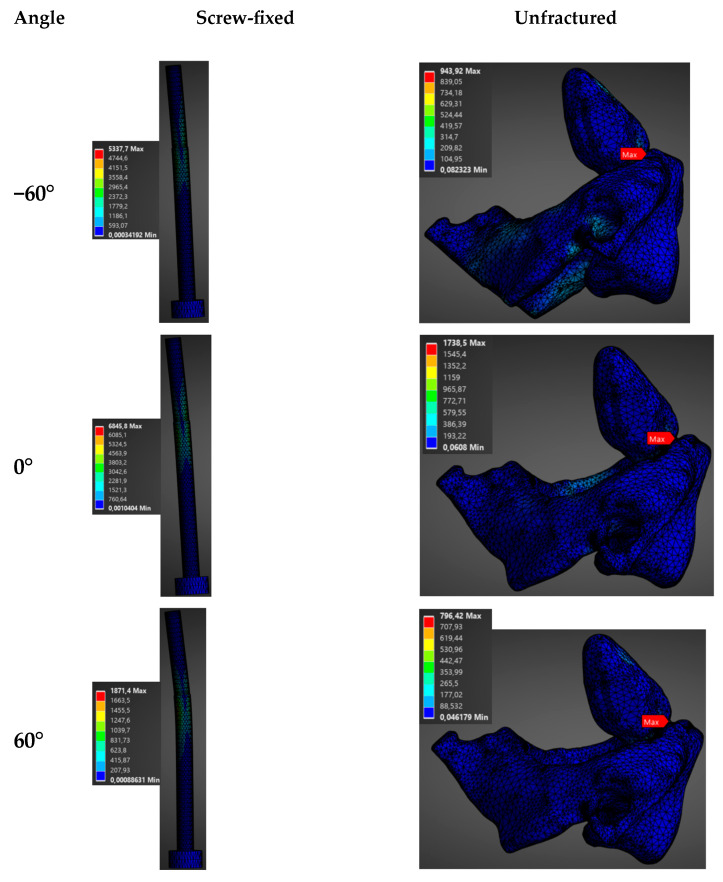
Distribution of stress (MPa) as a function of the load application angle (1500 N) in the sagittal plane for the unfractured and screw-fixed vertebrae.

**Figure 9:**
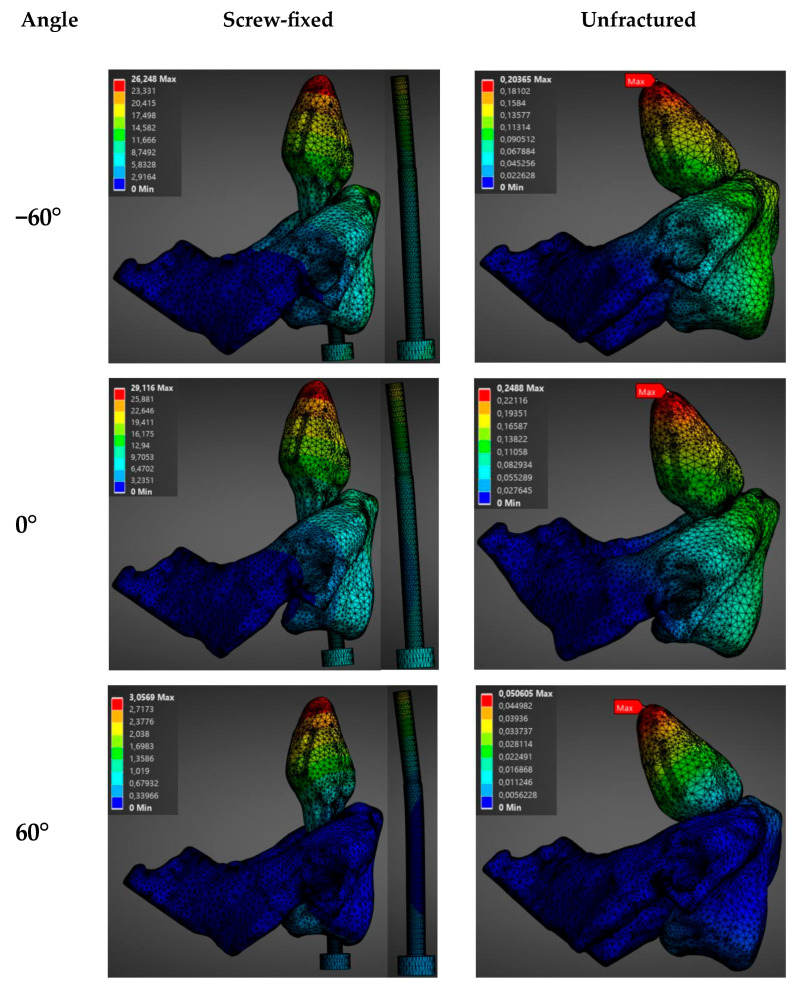
Distribution of total deformation (mm) as a function of the angle of application of a 1500 N load in the sagittal plane, for both the unfractured vertebra and the screw-fixed configuration.

The direction of the applied load had a notable impact on the biomechanics of the construct (see Figure 6, Figure 7, Figure 8 and Figure 9). Stresses and total deformations are, as in the previous case, always larger for the case of screw-fixed vertebra as compared to the normal vertebra (see Figure S3).

As noticed in Figure 6 and Figure S3a, the influence of the orientation of the load on the average stress is almost negligible when this is applied in the transversal plane. In this case, the values for the average stresses are almost constant for different angles of application. In this case, values around an average stress of 162.6 ± 10.7 MPa are observed for the screw-fixed vertebra, while a value of 63.5 ± 9.1 MPa is observed for the normal vertebra. With respect to the total deformation, no clear influence of the load application angle is observed when the load is applied in the transverse plane (see Figure 7 and Figure S3b).

On the contrary, a clear variation in the stresses and total deformations as a function of the angle of application is observed when the load is applied in the sagittal plane (see Figure 8 and Figure 9). In Figure S3 is observed that the maximum average stress and total deformation appear when the load is applied in the sagittal plane at an angle of 30°. In this case, a maximum stress of 179.39 MPa is observed at the screw, for the screw-fixed vertebra, and of 75.74 MPa at the base of the condyle for the normal vertebra. As in the previous case, the screw totally absorbed the load in the screw-fixed case.

### 3.3. Influence of the Sex

**Figure 10:**
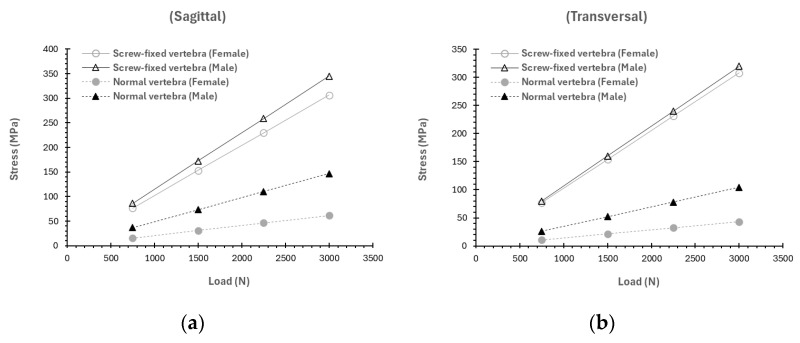
Variation as a function of the load and sex of the average stress (MPa) if the load is applied in the (**a**) sagittal plane (load direction at 0°) and in the (**b**) transversal plane (load direction at −60°).

**Figure 11:**
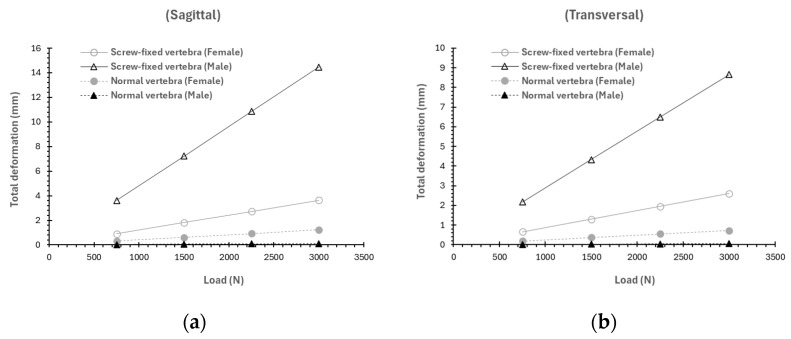
Variation as a function of the load and sex of the average total deformation (mm) if the load is applied in the (**a**) sagittal plane (load direction at 0°) and in the (**b**) transversal plane (load direction at −60°).

Biomechanical differences were observed between male and female models. Figure 10 and Figure 11 show stress and total deformation as a function of the load for both sexes. Under the same load magnitude and direction, the male models showed greater average stresses (both when the load is applied in the sagittal or transversal plane) for the screw-fixed cases (see Figure 10); however, when the load is applied in the transversal plane, the difference in average stress is negligible for the screw-fixed cases (Figure 10b). For the healthy cases, larger differences between male and female models are observed.

Similar results are observed with respect to total deformation. As seen in Figure 11, the average total deformation for the screw-fixed case is larger in males than in females; however, for the healthy cases, the deformation is larger in female than in male models, suggesting a greater structural vulnerability. This is supposed that can be likely explained by the lower bone cross-sectional area and density, and higher bone loss with age for females [34,35].

**Figure 12:**
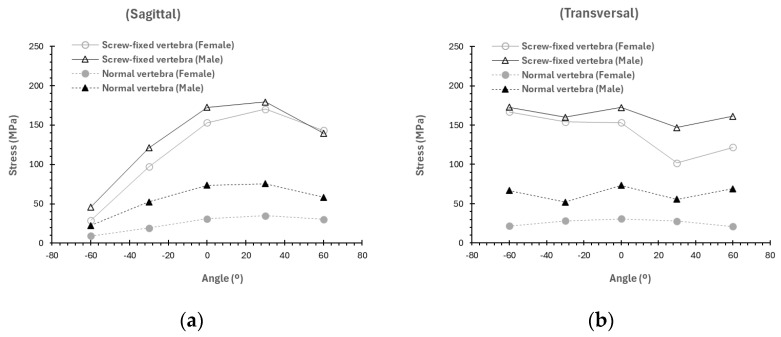
Variation as a function of the load and sex of the average stress (MPa) if the load is applied in the average stress if the load is applied in the (**a**) sagittal plane (load 1500 N), or in the (**b**) transversal plane (load 1500 N).

**Figure 13:**
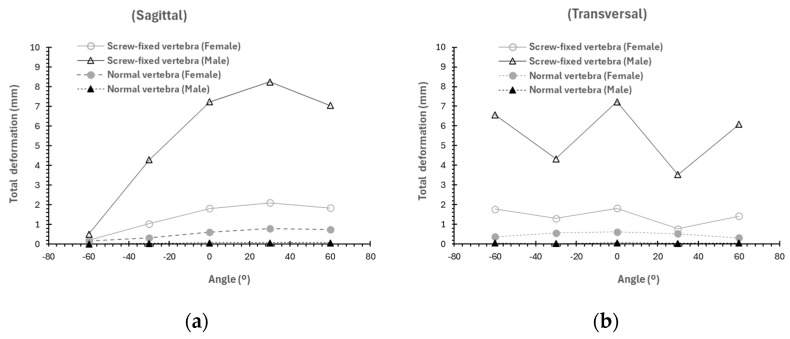
Variation as a function of the load and sex for the average total deformation (mm) if the load is applied in the (**a**) sagittal plane (load 1500 N) or in the (**b**) transversal plane (load 1500 N).

As observed in Figure 12 and Figure 13 the trends previously observed for the average stress and deformation repeat if we study the influence of the angle of application of the load (load fixed at 1500 N). Larger stresses are observed in male models compared to female models (regardless of whether the vertebra is screw-fixed or unfractured) when the load is applied in the sagittal plane rather than the transverse plane. Moreover, the maximum average stress is observed at a load application angle of 30° in the sagittal plane for both male and female models. If the load is applied in the transversal plane, the average stresses are independent of the direction of application of the load, except for the case of the screw-fixed model in the female model. In this case, a slight reduction in the value of stress is observed with the angle of application. Similar findings are found for the deformation (Figure 13).

## 4. Discussion

Values found in this study for the stress as a function of the magnitude and orientation of the load (Section 3.1 and Section 3.2) in unfractured models are in the same range and order of magnitude as those numerically determined by Scott Graham et al. [20] to predict the type of odontoid fracture. This confirms the validity of the model developed in this work, both in terms of properties and load application.

The results of this study highlight the stabilizing effect of anterior odontoid screw fixation in type II odontoid fractures under varying loading conditions. Anterior screw fixation significantly reduced both intervertebral displacement and bone stresses, especially under high axial load and oblique orientations—scenarios that replicate common clinical and traumatic situations. This behavior reinforces the indication of this technique in selected patients, particularly when preservation of atlantoaxial motion is desired [36].

The FEM analysis demonstrated that the magnitude of the applied force has a direct impact on the mechanical behavior of the system. As described in previous studies [37,38], progressive loading results in proportional increases in displacement and stress, which can compromise fracture site stability if adequate fixation is not achieved. Our findings confirm that the odontoid screw acts as a load-sharing component, absorbing part of the stress and reducing deformation in the surrounding bone tissue. This aligns with clinical observations of higher bone union rates in type II fractures treated with anterior fixation when anatomic alignment and direct axial compression are achieved.

Regarding load orientation, posterior oblique forces generate greater stress concentrations than vertical forces. This finding is especially relevant, as oblique forces often occur in secondary trauma (e.g., backward falls with cervical hyperextension) or even during daily activities in patients who are not fully recovered. Biomechanical studies by Wright et al. [39] and Panjabi et al. [40] had already noted that non-axial loads induce greater microdisplacement in unstable odontoid fractures; however, our study quantifies this difference under conditions of active screw fixation.

The comparison between male and female models revealed biomechanical differences consistent with the literature, which reports a greater structural vulnerability in females [41,42]. As seen in Figure 10b and Figure 12b, male models show the larger average stress; however, larger differences between male and female models are observed for the healthy cases. If we consider that women typically present lower bone density, greater trabecular porosity, and smaller geometric dimensions, this potentially results in a reduced capacity for load distribution and absorption. Moreover, in a surgical context, this difference between stresses in males and females may require the adjustment of the screw design (length, diameter) or the use of supplementary fixation, especially in elderly people with a history of osteoporosis, in order to avoid infra- or supra-dimensioned medical devices. These results suggest that FEM models could be used preoperatively to personalize surgical strategies according to patient-specific anatomy.

It should be emphasized that identical material properties were assigned to male and female models in order to isolate geometric effects. Therefore, the observed differences in stress and deformation are primarily attributable to anatomical morphology rather than to intrinsic differences in bone material properties. Future studies incorporating density-based material mapping from CT data may further elucidate the role of sex-specific bone quality.

The finite element method has been demonstrated as an effective tool for investigating cervical spine biomechanics [13]. Unlike cadaveric experimental models, which exhibit high variability and face ethical and logistical limitations, FEM models offer reproducibility, parametric control, and flexibility to simulate multiple clinical scenarios. Recent studies have extended their application to surgical planning, implant selection, and failure prediction [43,44,45,46]. In our study, the model allowed us to observe how small variations in load orientation or patient sex can modify stress distribution—data that would be difficult to quantify directly in real patients.

Among the main limitations of the present study is the model simplification: a homogeneous, isotropic linear elastic behavior was assumed for bone, without incorporating regional density variations or trabecular fiber orientation. Additionally, the model did not include ligamentous structures (for example, the transverse atlantal ligament), which may also contribute to atlantoaxial stability. Another limitation is that, although two representative morphologies by sex were analyzed, interindividual variability and the effects of comorbidities such as advanced osteoporosis were not considered. Nevertheless, we opted for a controlled approach that allowed isolation of key variables and clearer interpretation of the results.

Another limitation of our model is that it simplifies the C1–C2-Screw interface as bonded contacts; this does not take into account the tension of the “screw-bone interface,” so our models may over-stabilize the arthrodesis system.

Ultimately, this type of analysis may complement clinical expertise by helping to select ideal cases for anterior fixation, estimate the risk of mechanical failure, and propose more personalized treatment strategies. Since biomechanical behavior is closely linked to individual anatomy and cervical loading patterns, incorporating simulation techniques into surgical planning could improve functional outcomes and reduce complications.

## 5. Conclusions

The results of this biomechanical study, based on finite element simulations, confirm that anterior odontoid screw fixation provides substantial improvement in the mechanical stability of the C1–C2 segment in type II odontoid fractures compared to an unfixed fracture. This technique significantly reduces both intervertebral displacement and stress in the adjacent bone, particularly under high or oblique loading conditions, which are especially critical from a clinical perspective. We should also highlight that, despite the benefits provided by this screw fixation device, stress and deformation are always larger in screw-fixed cases than in unfractured ones.

It was demonstrated that the orientation of the applied force has a decisive influence on the distribution of stress and deformations, with posterior oblique loads generating the most unfavorable mechanical conditions. This underscores the importance of considering the predominant load vector involved in the fracture mechanism when selecting the most appropriate surgical strategy.

Additionally, relevant biomechanical differences were observed between male and female models, suggesting that anatomical and bone density factors should be considered during preoperative planning to optimize surgical outcomes and prevent implant-related mechanical failures.

This study contributes to a better understanding of the biomechanical variables involved in the surgical treatment of this cervical fracture and highlights the value of FEM models as a complementary tool in clinical decision-making.

## Figures and Tables

**Table 1:** Mechanical properties of the materials used in the FEM model.

Material	Young’s Modulus (MPa)	Poisson’s Ratio
Cortical bone [28,29]	12,000	0.30
Cancellous bone [29,30]	450	0.20
Ti6Al4V ELI [31,32]	96,000	0.36

## Data Availability

Dataset available on request from the authors.

## Supplementary Materials

The following supporting information can be downloaded at: https://www.mdpi.com/article/10.3390/ma19040825/s1, Table S1: Results of mesh sensitivity analysis for an unfractured vertebra. Figure S1: Schematic representation showing the applied load and fixed surfaces on a representative model for the (a) normal and the (b) screw-fixed vertebras. Figure S2: Variation as a function of the load (applied in the sagittal-load at 0° and transversal plane-load at 60°) for the average (a) stresses and (b) deformation in the screw-fixed and in the unfractured vertebra. Figure S3: Variation as a function of the direction of the load (applied in the sagittal and transversal planes) for the average (a) stress and (b) deformation in the screw-fixed and in the unfractured vertebra. Supplementary Video S1: Deformation-Screw Fixed Vertebra. Supplementary Video S2: Unfractured Vertebra.
