# Supplementary material for: Finite Element Analysis of Anterior Odontoid Screw Fixation for Type II Odontoid Fractures
Source: Materials (Basel). 2026 Feb 23;19(4):825. doi: 10.3390/ma19040825 (PMC12942386; doi:10.3390/ma19040825)
Supplement: Supplementary file 1 [file materials-19-00825-s001.zip › materials-4095396-supplementary.pdf]

materials

MDPI

Supplementary

Finite Element Analysis of Anterior Odontoid Screw Fixation for Type II Odontoid Fractures

Pedro Miguel González-Vargas *, Antía Millán, José Luis Thenier-Villa, Aida Badaoui, Cesáreo Conde, Juan Pou and Antonio Riveiro *

Table S1. Results of mesh sensitivity analysis for an unfractured vertebra.

Mesh Size (mm)	No. of Nodes	No. of Elements	Average Von Mises Equivalent Stress (MPa)	Average Elastic Strain (mm)	Screw Displacement (mm)
5	88992	52168	47.685	3.98×10^{-2}	2.18×10^{-2}
2	89392	52446	47.392	3.96×10^{-2}	2.16×10^{-2}
1.7	89255	52242	47.477	3.95×10^{-2}	2.14×10^{-2}
1.4	91205	53416	46.941	3.94×10^{-2}	2.12×10^{-2}

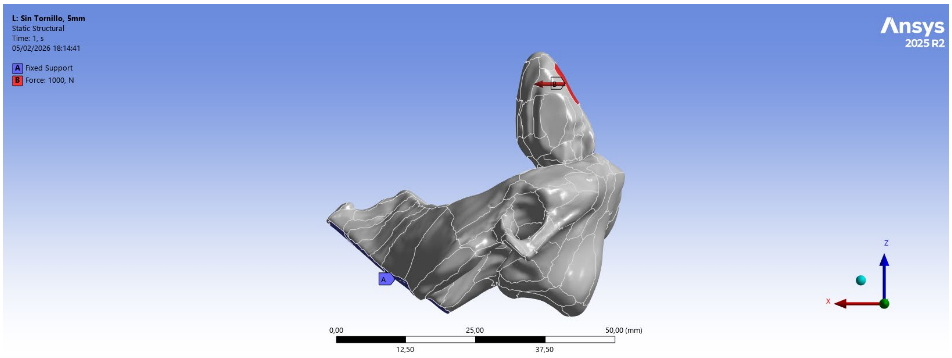

(a)

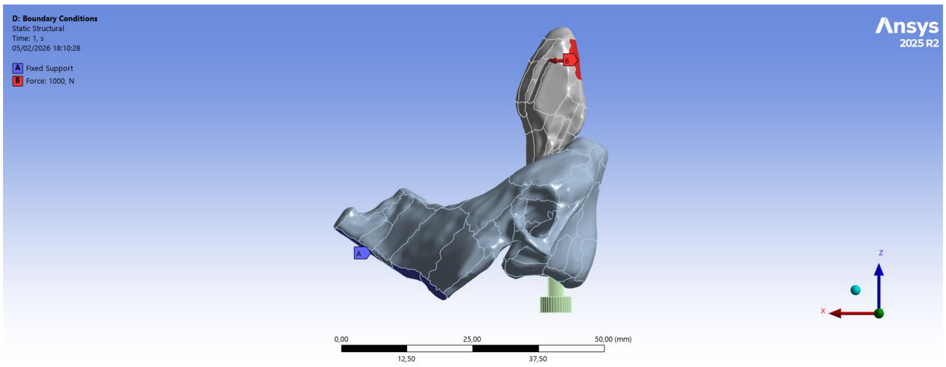

(b)

Figure S1. Schematic representation showing the applied load and fixed surfaces on a representative model for the (**a**) normal and the (**b**) screw-fixed vertebra.

 https://doi.org/10.3390/ma19040825

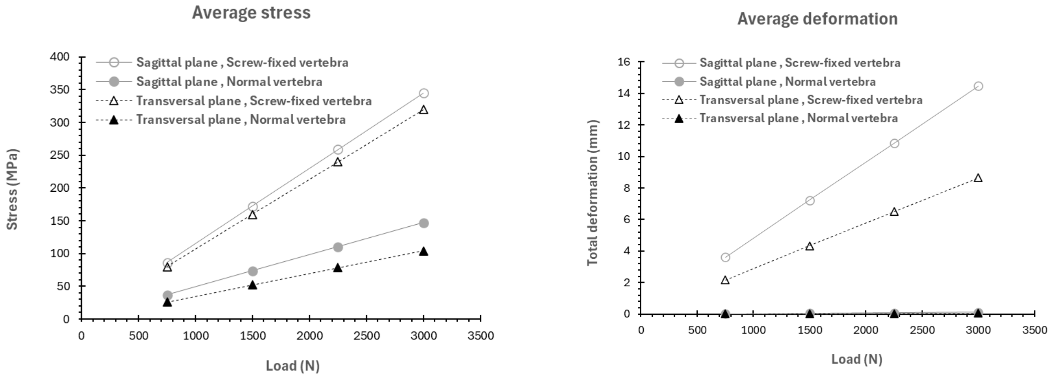

(a) **(b)**

Figure S2. Variation as a function of the load (applied in the sagittal-load at 0º and transversal plane-load at 60º) for the average (**a**) stress (MPa) and (**b**) deformation (mm) in the screw-fixed and in the unfractured vertebra.

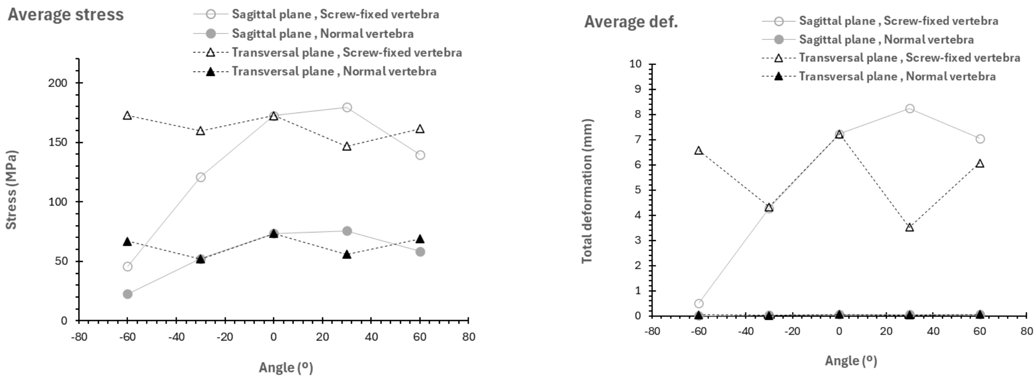

(a) **(b)**

Figure S3. Variation as a function of the direction of the load (applied in the sagittal and transversal planes) for the average (**a**) stress (MPa) and (**b**) deformation (mm) in the screw-fixed and in the unfractured vertebra.

https://doi.org/10.3390/ma19040825
